# Nanomaterials in Water Applications: Adsorbing Materials for Fluoride Removal

**DOI:** 10.3390/nano11071866

**Published:** 2021-07-20

**Authors:** Lei Huang, Kuilin Wan, Jia Yan, Lei Wang, Qian Li, Huabin Chen, Hongguo Zhang, Tangfu Xiao

**Affiliations:** 1 School of Environmental Science and Engineering, Guangzhou University, Guangzhou 510006, China; huanglei@gzhu.edu.cn (L.H.); klwan1996@163.com (K.W.); jiayan@gzhu.edu.cn (J.Y.); dreamboatrn@163.com (L.W.); qianli@gzhu.edu.cn (Q.L.); xun1597820419@163.com (H.C.); tfxiao@gzhu.edu.cn (T.X.); 2 Guangzhou University-Linköping University Research Center on Urban Sustainable Development, Guangzhou University, Guangzhou 510006, China; 3 State Key Laboratory of Geohazard Prevention and Geoenvironment Protection, Chengdu University of Technology, Chengdu 610059, China

Fluoride is an important pollutant in many countries, such as China, India, Australia, the United States, Ethiopia, etc. Too low concentrations of fluoride cause osteoporosis and saprodontia, leading to the use of toothpaste with fluoride. However, more regions have higher concentrations of fluoride than is needed due to dry weather and geological conditions, especially in industries containing fluoride pollution. The drinking water standard of fluoride is ruled by World Health Organization as 1.5 ppm, and the regulated standard in China is 1.0 ppm [1]. Taking in too much fluoride during a long period brings about Ayers’ disease, fluorosis of bone, dental fluorosis, kidney stone, intestinal and liver disorders, etc. Therefore, different treatment technologies are investigated to deal with excessive fluoride.

C. S. Boruff reported the treatment of wastewater containing fluoride by using sufficient calcium hydroxide to precipitate in January 1934 [2]. The precipitation method opens the floodgates to remove fluoride. Adsorption, ion exchange, electric flocculation, membrane technology, solvent extraction, and electro-adsorption were applied to remove fluoride from nature, life, and industries [3,4,5,6,7,8,9,10]. Ion exchange needs ion-exchange resin, which makes it easy to exchange fluoride in an ion exchange column. However, the ion-exchange resin is easy to reach saturation and often needs to regenerate. Electric flocculation makes use of electrical energy that could change metal to metal ions. The metal ions could combine fluoride to bring about flocculation. It brings metallic contamination and power consumption. Fluoride can be obstructed by the pore diameter of a membrane. Membrane fouling is an important risk to the technology. Solvent extraction requires extraction and reverse extraction. The redundant process limits the application.

Adsorption and electro-adsorption use the bonding ability of materials with fluoride. Adsorption is also an important way of dealing with water pollution [11]. Electro-adsorption is the development of adsorption that applies an electric field to enhance the binding capacity of materials for removing fluoride. Adsorbing materials are the dominant factor for improving the adsorption capacity, adsorption rate, high selectivity, range of pH, price, and recycling property. In this paper, we will discuss the adsorbing material of fluoride due to it being most researched in this field This paper covers: (1) the past of adsorbing material from 1930 to 2000: the initial preparation, application for removing fluoride; (2) the present of adsorbing material from 2001 to 2021: modified, mechanism about fluoride removal; (3) the future for developing adsorbents: design, screen for capturing fluoride. This provides the timeline of the development of adsorbing material for dealing with wastewater containing fluoride.

The past of adsorbing material: Ralph H. McKee and William S. Johnston reported that four different carbons were applied to remove fluoride, and some kinds of carbons were more efficient and promising [12]. C. S. Boruff also carried out similar work by using contact beds with the activated alumina or aluminum compounds [2]. This research provided a good attempt and guidance for the possibility of adsorbing fluoride. G. J. FINK and F. K. LINDSAY used Al_2_O_3_ to adsorb fluoride, and the adsorption capacity could decline with the increase in pH. Others research proved that the addition of magnesium may improve the ability to remove fluoride [13]. Then, many researchers investigated alumina, until now. At present, aluminium oxide is recommended as the optimizing and commercial sorbent by the World Health Organization. H. Farrah and W. F. Pickering reported hydrous iron oxides [14], Toshishige M. SUZUKI applied hydrous zirconium oxide and La/chelating resin [15,16], Chong Mou Wang and Thomas E. Mallouk investigated titanium dioxide [17], and J. Nomura, H. Imai and T. Miyake used hydrous cerium oxide [18] to remove fluoride from wastewater. In this stage, most adsorbing materials were immediately purchased or simply prepared to find the possibility of fluoride removal. This period offered the fundaments and inspiration for the subsequent research in adsorbing fluoride.

The present of adsorbing material: with the development of industry and society, high concentrations of, and complex wastewater containing fluoride need to be disposed. Hence, biomass, thallus, nano-metal oxide, graphene, metal organic frameworks, carbon nanotubes, polymers, layered double hydroxides, and perovskite are applied to improve the performances of removing fluoride [19,20,21,22,23,24,25,26,27]. There are also other different adsorbing materials for fluoride removal. The developments of adsorptive directions are summarized as: (A) finding new kinds of materials: Sn(II)-TMA metal organic framework (MOF) demonstrated efficient adsorption efficiencies at a wide pH in simulated wastewater [28]; (B) modifying adsorbing materials: Fe_3_O_4_/γ-MnO_2_ meso-porous nanocomposite furnished adsorption sites O-Mn-OH to exchange -OH with -F. The study offered an effective modified approach [29]; (C) density functional theory: intrinsic, B-doped, and Al-doped graphene were revealed to affect the adsorption changes of F^−^ ions and HF molecules. Among them, the Al-doped graphene was more easily combined with F^−^ ions. The molecule of HF could be only chemisorbed on Al-doped graphene [30]; (D) complicated wastewater: Acinetobacter H12 was investigated to deal with wastewater containing calcium, fluoride, and nitrate [31]. Layered double oxides were studied for remediating the industrial wastewater containing manganese and fluoride [32]; (E) technological upgrade: Ti(OH)_4_/activated carbon was used as the electrode to remove fluoride that can reach 115.2 mg/g when a voltage of +1.2 V was investigated and regenerated quickly at −1.6 V [33].

The future for developing adsorbents: There were more efforts to understand the process of fluoride clearly. (1): Mechanisms and models: the mechanism of fluoride can be revealed more clearly with the development of materials’ characterization and theoretical calculation, such as synchrotron radiation source, cryoelectron microscopy, etc. The in-depth mechanism can generate adsorption models for removing fluoride; (2): electrosorption: this newly developed technology will have caused more interest. Many problems need to be further investigated; (3): complicated wastewater: wastewater contains too many constituents for modern society. The high selectivity and specificity of fluoride are one goal for complicated wastewater. What is more, the simultaneous adsorption of contaminants is also another goal; (4): machine learning and artificial intelligence will be important tools to design the adsorbing materials. They offer an efficient and goal-oriented way to sieve adsorbing materials and modified crystal structures; (5): advanced materials: the advanced materials will open a new possibility to exhibit new series adsorbing materials, for example, MXene, graphdiyne, etc. These design ideas will be applied to modify advanced materials to improve the capability of fluoride removal.

Humans and nature are finding the equilibrium between fluoride and health. It requires the discovery of new technology and material. Urbanization keeps the urban drinking water lower than the fluoride drinking standard of 1 ppm. However, rural drinking water is still confronted with a great challenge from industrial urban development. It should utilize the resource of fluoride. This Special Issue is built to promote the researchers who deliver these thoughts, ideas, and discoveries of nanomaterials in water applications. Thank you to everyone who wants to, or can contribute to this Special Issue.

## Data Availability

Data sharing not applicable.
